# Association of glycogen synthase kinase-3β with Parkinson’s disease (Review)

**DOI:** 10.3892/mmr.2014.2080

**Published:** 2014-03-28

**Authors:** DA-WEI LI, ZHI-QIANG LIU, WEI-CHEN, MIN-YAO, GUANG-REN LI

**Affiliations:** 1 Department of Neurology, Affiliated Hospital of Beihua University, Jilin, Jilin 132000, P.R. China; 2 Department of Neurology, The First Hospital of Jilin University, Changchun, Jilin 130021, P.R. China; 3 Department of Neurology, The Third Hospital of Jilin University, Changchun, Jilin 130021, P.R. China

**Keywords:** Parkinson’s disease, glycogen synthase kinase-3β, regulation

## Abstract

Glycogen synthase kinase-3 (GSK-3) is a pleiotropic serine/threonine protein kinase found in almost all eukaryotes. It is structurally highly conserved and has been identified as a multifaceted enzyme affecting a wide range of biological functions, including gene expression and cellular processes. There are two closely related isoforms of GSK-3; GSK-3α and GSK-3β. The latter appears to play crucial roles in regulating the pathogenesis of diverse diseases, including neurodegenerative disease. The present review focuses on the involvement of this protein in Parkinson’s disease (PD), a common neurodegenerative disorder characterized by the gradually progressive and selective loss of dopaminergic neurons, and by intracellular inclusions known as Lewy bodies (LBs) expressed in surviving neurons of the substantia nigra (SN). GSK-3β is involved in multiple signaling pathways and has several phosphorylation targets. Numerous apoptotic conditions can be facilitated by the GSK-3β signaling pathways. Studies have shown that GSK-3β inhibition protects the dopaminergic neurons from various stress-induced injuries, indicating the involvement of GSK-3β in PD pathogenesis. However, the underlying mechanisms of the protective effect of GSK-3β inhibition on dopaminergic neurons in PD is not completely understood. Multiple pathological events have been recognized to be responsible for the loss of dopaminergic neurons in PD, including mitochondrial dysfunction, oxidative stress, protein aggregation and neuroinflammation. The present review stresses the regulatory roles of GSK-3β in these events and in dopaminergic neuron degeneration, in an attempt to gain an improved understanding of the underlying mechanisms and to provide a potential effective therapeutic target for PD.

## 1. Introduction

Parkinson’s disease (PD) is a common neurodegenerative disorder characterized by the gradually progressive and selective loss of dopaminergic neurons and by intracellular inclusions known as Lewy bodies (LBs) expressed in the surviving neurons of the substantia nigra (SN) (1). The progressive loss of dopaminergic neurons is a complex process, and multiple pathological events, including oxidative stress, mitochondrial dysfunction, protein aggregation and neuroinflammation, are indicated in PD pathogenesis (2,3). Substantial evidence indicates that mitochondrial dysfunction induced by diverse stress conditions plays a crucial role in the pathogenesis of PD (4,5). A central event in the mitochondrial cell death pathway is the formation of a mitochondrial permeability transition pore (mPTP). The production of reactive oxygen species (ROS) triggered by complex I inhibition are believed to be key inducers of mPTP formation (6). Complex I deficiency has been shown to contribute to the dopaminergic cell death in idiopathic PD patients (7,8). The administration of 1-methyl-4-phenyl-1,2,3,6-tetrahydropyridine (MPTP) and rotenone, well-known inhibitors of complex I, induce PD syndrome characterized by the loss of SN neurons in animal models (9–11), supporting the involvement of mitochondrial dysfunction in the pathogenesis of PD. Decreased complex I activity in the mitochondrial respiratory chain leads to excessive ROS production, which contributes to the oxidative damage of cellular macromolecules and the activation of mPTP, ultimately leading to cell death (12,13). Neuroinflammation has increasingly been recognized as a pathological contributor to neurodegenerative diseases (14–16), and particularly as a key promoter to the chronic loss of nigral dopaminergic neurons in PD (17). Postmortem studies revealed activated microglia and accumulation of inflammatory mediators expressed in the SN of PD patients and animal models (16,18,19). Inhibition of the inflammatory response promotes dopaminergic neuron survival in various PD models (20–22), confirming the indicated action of the inflammatory response in neurodegenerative diseases. Several studies have revealed that the inhibition of GSK-3β reduces dopaminergic neuron injury induced by MPTP toxicity, indicating the association of GSK-3β with the pathogenesis of PD (23,24). GSK-3β is a central point in a number of signaling pathways in the pathogenesis of this neurodegenerative disease, affecting multiple pathological events involved in dopaminergic neuron degeneration, thus providing a potential target in the therapeutic management by blocking the pathogenic pathways involved in PD pathogenesis.

## 2. Properties of GSK-3

**Figure 1:**
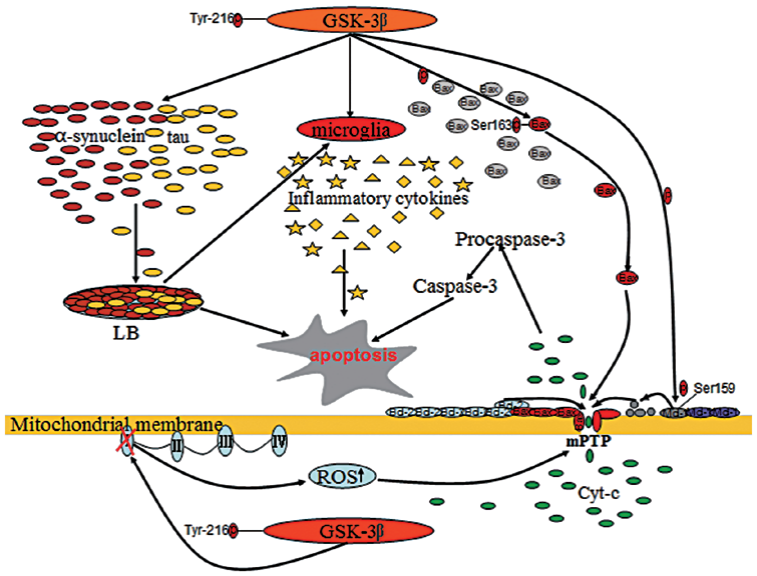
Glycogen synthase kinase-3β (GSK-3β) facilitates the toxic effects of mitochondrial dysfunction, protein aggregation and inflammatory response on dopaminergic neurons. Mitochondrial GSK-3β inhibits complex I activity thus increasing reactive oxygen species (ROS) production. This production of ROS contributes to the oxidative damage of cellular macromolecules, including proteins, lipids and DNA, and facilitates mitochondrial permeability transition pore (mPTP) formation. Cytosolic GSK-3β phosphorylates the α-synuclein and τ proteins, leading to their aggregation, which contributes to cell injury by oxidative stress and the inflammatory response. Activated GSK-3β can also promote the inflammatory response by activating microglia and increasing the production of inflammatory cytokines. In addition, the GSK-3β signal upregulates the levels of Bax and promotes its mitochondrial membrane translocation. Once located in the membrane, this protein increases the mitochondrial membrane permeabilization by sequestering Bcl-2 and oligomerization, finally causing the release of cytochrome *c* and cell death. Additionally, GSK-3β phosphorylates Mcl-1 on Ser159, resulting in the destabilization of this protein and blockage of the Mcl-1 dependent integrity of the mitochondrial membrane. Cyt-c, cytochrome *c*; LB, Lewy body; Ser, serine; Bcl, B-cell lymphoma.

GSK-3 is a serine/threonine (Ser/Thr) protein kinase expressed in the cytosol, nucleus and mitochondria of all eukaryotic cells. There are two major GSK-3 protein isoforms (GSK3α and GSK3β) encoded by two highly homologous genes, gsk-3α and gsk-3β (25). This enzyme was originally identified as a regulator of glycogen synthase (26). However, GSK-3 has been recognized as a pleiotropic enzyme, affecting numerous biological functions including gene expression and cellular processes such as cell proliferation, differentiation and apoptosis (27,28). GSK-3 phosphorylates and regulates >50 substrates, which allows this enzyme to modulate a wide range of biological functions (27,28). Dysregulation of GSK-3 is implicated in diverse diseases, including diabetes, ischemia/reperfusion injury, bipolar disorder, cancer and neurodegenerative disease (27,29–32). GSK-3β is a point of convergence for multiple signaling pathways and thus plays a crucial role in regulating the pathogenesis of diverse diseases. Its activity and functions are controlled by phosphorylation at specific sites. Phosphorylation at Ser9 of GSK-3β markedly inhibits its activity (33), whereas phosphorylation at Tyr216 increases its activity. The inactivation of GSK-3β is mainly targeted by the Akt signaling pathway by the phosphorylation of Ser9 of this enzyme. GSK-3β is mainly localized in the cytosol, but lower amounts are expressed in the nucleus and mitochondria (34–37), and its regulatory role in the mitochondrial cell death pathway has been elicited by a variety of stress conditions shown in neuronal cells (38–43). GSK-3β facilitates numerous apoptotic conditions involved in PD pathogenesis, including mitochondrial dysfunction, oxidative stress, protein aggregation and the inflammatory response, by modulating diverse signaling pathways (Fig. 1) (23,24,42). Inhibition of GSK-3β is indicated in the suppression of a number of pathogenic events in PD, thus promoting dopaminergic neuronal survival (23,24,44,45).

## 3. Regulation of GSK-3β in mitochondrial complex I activity and ROS formation

In eukaryotic cells, mitochondria are key organelles providing essential energy for cell metabolism through adenosine triphosphate (ATP) generation. Complex I is a protein component of the electron transport chain located in the inner part of the mitochondrial membrane and functioning as the effective enzyme of the oxidative phosphorylation system responsible for the generation of cellular ATP. Mitochondrial complex I is the main site of ROS formation as it transfers single electrons to oxygen, thus generating O_2_^−^ and subsequently H_2_O_2_ (46,47). Inhibition of complex I leads to a decrease in ATP levels and excessive production of ROS, the central events of mitochondrial dysfunction that have been indicated in PD pathogenesis (48). The first evidence for the involvement of complex I inhibition in PD was the recognition that MPTP caused a severe and irreversible parkinsonian syndrome in drug abusers (49). MPTP is a lipophilic molecule and can rapidly cross the blood-brain barrier. Once it crosses the barrier, it is oxidized in the brain to its toxic metabolite 1-methyl-4-phenylpyridinium (MPP^+^) by type B monoamine oxidase (50). MPP^+^ is then taken up by dopaminergic neurons via a dopamine transporter and accumulates in the mitochondria where it causes excessive ROS formation by inhibiting respiration complex I (51), finally leading to dopaminergic neuron death. Mitochondrial complex I inhibition has also been reported in the SN, platelets and skeletal muscle of idiopathic PD patients (7,8,52). Complex I is generally known to be the primary source of mitochondrial ROS (53–55). Inhibition of mitochondrial complex I elicited by neurotoxins MPP^+^ and rotenone, well-established dopaminergic cell death inducers in PD, have been shown to increase the production of ROS (5,56). GSK-3β has been shown to be located into the mitochondria, where it is highly activated compared with the cytosolic form (36). Although the significance of the presence of GSK-3β in the mitochondria remains poorly understood, its involvement in mitochondrial dysfunction has been reported (57,58). GSK-3β can regulate cell survival and apoptosis by controlling mitochondrial complex I activity and ROS production (43). GSK-3β regulates oxidative phosphorylation by inhibiting NADH (complex I), which is the main site of ROS formation, whereas this enzyme is implicated in homeostatic redox equilibrium (43). Previous studies have shown that mitochondrial toxins, including rotenone and MPTP treatments, increase GSK3β activity. Inhibition of GSK-3β protects dopaminergic neurons from the toxicity of rotenone and MPTP, indicating the involvement of GSK-3β in the complex I inhibition-induced cell death pathway in PD (23). Studies in the MPTP model of PD also demonstrate that mitochondrial GSK-3β significantly promotes ROS production by further inhibiting complex I, and that this can be reversed by GSK-3β inhibitors (43). Similar studies have indicated that GSK-3β inhibition promotes mitochondrial biogenesis and prevents ROS production during ischemic cerebral damage (59). Although the mechanism underlying the contribution of GSK-3β to the mitochondrial complex I inhibition remains unclear, these reports clearly indicate that GSK-3β inhibition contributes to cell survival induced by mitochondrial complex I inhibition and ROS formation.

## 4. GSK-3β and mitochondrial intrinsic apoptosis pathway

Mitochondria are integrated in diverse signaling pathways linked to multiple cell processes, including apoptosis. The major property for mitochondria is the maintenance of its membrane potential and the low-conductance state of the mitochondrial permeability transition pore (mPTP) in living cells. mPTP activation is a central event in mitochondria-mediated intrinsic cell apoptosis, which has been implicated in the pathogenesis of PD and several other neurodegenerative disorders (60–62). The mPTP pathway of cell death is mediated by the disruption of the mitochondrial membrane and the release of apoptogenic molecules, which can be regulated by GSK-3β signaling pathways through modulating the opening of the mPTP (63,64). Studies have shown that GSK-3β inactivation protects cardiac cells from ischemia/reperfusion injury through the inhibition of the mPTP opening, indicating its regulatory role in the mitochondrial cell death pathway (65–68). It has also been shown in cell and animal models of PD that GSK-3β inhibition can protect dopaminergic neurons from MPTP toxicity (23,24,42,69). This contribution of GSK-3β to cell death and survival appears to correlate with its ability to control the mitochondrial localization and activation of a number of proteins, particularly B-cell lymphoma 2 (Bcl-2) family proteins, including Bax, Bcl-2 and Mcl-1, considered as central players in mPTP formation (70,71). Generally, Bax is a cytosolic protein that can be translocated to the mitochondrial membrane in response to apoptotic stimuli (72). Once located in the mitochondrial membrane, this protein increases the mitochondrial membrane permeabilization by sequestering Bcl-2 and by oligomerization within the mitochondrial membrane, leading to the release of pro-apoptotic molecules into the cytoplasm (73,74). By contrast, Bcl-2 and Mcl-1 are anti-apoptotic members that preserve mitochondrial membrane integrity, thereby preventing the release of apoptogenic molecules and cell apoptosis (75). GSK-3β activation promotes mitochondria-mediated apoptosis by the upregulation of Bax expression levels (58,76). Treatment with lithium, a pharmacological inhibitor of GSK-3β, could suppress the pro-apoptotic pathway by decreasing the expression levels of Bax, but promote anti-apoptotic signaling through increasing Bcl-2 expression (77–79). In addition, GSK-3β can facilitate the mitochondrial localization of Bax by directly phosphorylating Ser163 of this protein (70). In PD, models reveal that the inhibition of GSK-3β protects dopaminergic cells against neurotoxin-induced damage through attenuating the translocation of Bax to the mitochondria (80–82). Additionally, GSK-3β phosphorylates Mcl-1 on Ser159, resulting in the destabilization of this protein and the blockage of the Mcl-1-dependent integrity of the mitochondrial membrane (71). Overall, GSK-3β may be vital in the regulation of cell death and survival through the modulation of the mitochondrial apoptotic cell death pathway.

## 5. Regulation of GSK-3β in α-synuclein and τ protein expression and aggregation

Protein aggregation and inclusion body formation in selected areas of the neuronal system are pathological hallmarks of neurodegenerative diseases, including PD, in which intracellular inclusions known as LBs are expressed in surviving SN neurons. LBs are composed mainly of the α-synuclein protein, a presynaptic neuronal protein abundantly expressed in the nervous system (83–85). The regulatory role of α-synuclein in the production of dopamine through the interaction with tyrosine hydroxylase has been shown in cultured cells (86,87). TH is the rate-limiting enzyme responsible for the conversion of tyrosine to L-3,4-dihydroxyphenylalanine in the dopamine synthesis pathway (86,88). Overexpression of α-synuclein inhibits TH activity and decreases dopamine biosynthesis, while suppression of α-synuclein expression levels promotes TH activity and consequently increases dopamine production (86,87,89). The toxicity of α-synuclein overexpression and accumulation to the neurons has been established in *in vivo* and *in vitro* models (90–93). α-synuclein protein overexpression and aggregation exacerbate the impairment of mitochondrial functions by augmenting oxidative stress (94–97). This protein overexpression can also directly activate microglia via a classical activation pathway, leading to the increase of the inflammatory response by the production and release of proinflammatory mediators (98–100). The actions of α-synuclein in promoting oxidative stress and the inflammatory response may be the underlying mechanism responsible for the toxicity of its overexpression and accumulation to dopaminergic neurons in PD. α-synuclein is a substrate for GSK-3β phosphorylation. GSK-3β inhibition decreases α-synuclein protein expression and prevents cell death in a cellular model of PD, indicating that inhibition of GSK-3β activity may be neuroprotective to dopaminergic neurons by attenuating the toxicity of α-synuclein overexpression (101). τ protein was originally discovered as a key component of intracellular neurofibrillary tangles within the brain of AD patients, however, this protein is also expressed highly in LBs and in the striatum of PD brains, indicating that it contributes to the pathogenesis of PD (102,103). Blockage of τ phosphorylation with special inhibitors prevents the dopaminergic neuronal death of PD models (101). GSK-3β is a main kinase affecting τ function through interfering with τ phosphorylation. Activation of GSK-3β increases τ phosphorylation (104–106), which can be reversed by GSK-3β inhibitors or upstream Akt inhibitors (107,108). Additionally, GSK-3β may also facilitate the aggregation of τ protein and neurodegeneration (109,110). Animal models indicate that the inhibition of GSK-3β promotes neuron survival by reducing τ-induced toxicity (111–113). These findings provide a potential target in the therapeutic management of PD by blocking the pathogenic pathway of protein overexpression and aggregation.

## 6. GSK-3β and neuroinflammation

**Figure 2:**
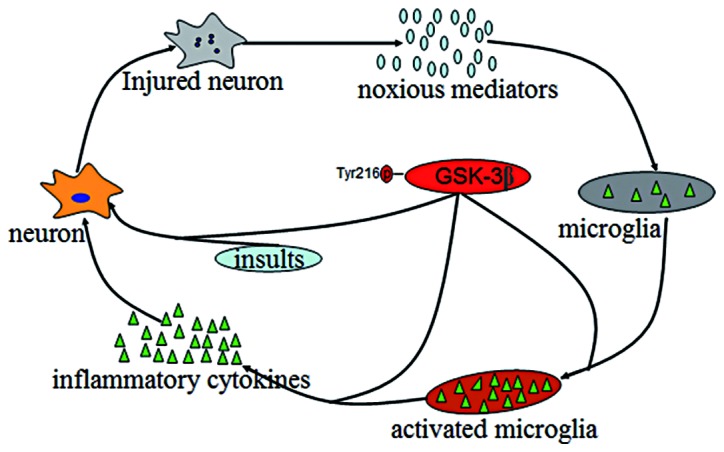
Glycogen synthase kinase-3β (GSK-3β) signaling pathways promote an uncontrolled, prolonged inflammatory and neuron injury process in Parkinson’s disease (PD). GSK-3β can facilitate multiple insult-induced neuronal injuries, thus generating a spectrum of noxious endogenous mediators, which contribute to the activation of microglia. Activated microglia produce and release proinflammatory cytokines, which can be promoted by GSK-3β signaling pathways, resulting in further neuron damage by the inflammatory response through oxidative stress and cytokine toxicity. Thereby GSK-3β plays a central role in the maintenance of the vicious cycle between neuron damage and microglia activation, leading to an uncontrolled, prolonged inflammatory and neuron injury process.

The inflammatory response, including a host of cytokines has been shown to be implicated in neuronal degeneration in PD and other neurodegenerative diseases (15,114). The activation of microglia and the upregulation of proinflammatory cytokines are key characters of brain inflammation. Microglia are resident immunocompetent cells in the brain and become activated in response to infection and damage (115). The release of proinflammatory and neurotoxic mediators from activated microglia contributes to progressive neuron damage in neurodenerative conditions (116,117). Studies have shown that microglia are activated regionally in the SN of PD patients and animal models (16,18,19,118), and that the levels of a number of inflammatory cytokines, including tumor necrosis factor-α (TNF-α), interleukin (IL)-1β, IL-2 and IL-6, are also upregulated in PD (119–122), indicating the involvement of the inflammatory response in PD pathogenesis. The contribution of inflammation-derived oxidative stress and cytokine-dependent toxicity to the nigrostriatal dopaminergic neuron death has also been reported in PD models (117,123,124). Additionally, suppression of the inflammatory response leads to the protection of dopaminergic neurons against neurotoxin-induced cell damage (22,125), which further supports the indication that the inflammatory mechanism is involved in neurodegenerative disease. Microglia can be activated by injured neurons through generating a spectrum of noxious endogenous mediators. Once activated, microglia produce and release multiple proinflammatory factors. This production of proinflammatory factors in turn exacerbates neuron damage by oxidative stress and cytokine toxicity (14,19), leading to further release of noxious endogenous mediators from injured neurons and an everlasting inflammatory response. This positive feedback between activated microglia and damaged neurons contributes to an uncontrolled, prolonged inflammatory process, which is believed to be, at least in part, responsible for the progressive loss of dopaminergic neurons in PD (17,114). Thereby, inhibition of the inflammatory response caused by microglia activation may be beneficial in neurodegenerative conditions. GSK-3β is a point of convergence of a wide range of signaling pathways, and has been recognized as a key regulator of inflammation (126,127). Activation of GSK-3β promotes inflammatory responses by activating microglia and increasing the production of inflammatory cytokines (45,128–129). The signals of GSK-3β can also promote various insult-induced neuronal injuries and the noxious generation of endogenous mediators. Thus, GSK-3β plays a central role in the maintenance of the vicious cycle between activated microglia and damaged neurons responsible for the progressive loss of dopaminergic cell loss in PD (Fig. 2). Inhibition of GSK-3β attenuates the microglia response to inflammatory stimuli and reduces cytokine production, thereby providing protection from inflammation-induced toxicity (45,126). However, the direct substrates of GSK-3β that are involved in inflammation-induced neuron damage remain unclear. TNF-α may be a key downstream signal transducer that is indicated in the proinflammatory effect of GSK-3β in activated microglia-mediated neuroinflammation (130). Within the brain, TNF-α is a mainly proinflammatory cytokine that is released by activated microglia in response to various insults or injury. This production of TNF-α triggers the uncontrolled inflammatory response by further activating microglia (131), which can be blocked by GSK-3β inhibition through modulation of nuclear factor κB and mixed lineage kinase 3/c-Jun N-terminal kinase 3 signaling cascades (130). These findings indicate that GSK-3β activity is critical for neuronal death in response to the neuroinflammation elicited by the microglial activation. Attenuation of the microglia-mediated inflammatory response targeted by GSK-3β inhibition to prevent dopaminergic neuron degeneration in PD requires further investigation.

## Conclusion

The pathogenesis of PD is a complex process, and multiple pathological events, including oxidative stress, mitochondrial dysfunction, protein aggregation and neuroinflammation, are considered to mediate and drive the gradual loss of dopaminergic neurons in PD (8,132–134). Understanding the intracellular signaling processes that regulate the events involved in the pathogenesis of PD is critical for developing novel therapeutics for PD treatment. GSK-3β is a multifaceted enzyme that has been indicated to be involved in the pathogenesis of neurodegenerative diseases, including PD, by modulating multiple signaling pathways (101,135–137). GSK-3β inhibition protects dopaminergic neurons from various stress-induced injuries in the cell culture and animal models of PD (23,42). The cellular and molecular mechanisms of the protective effects of GSK-3β inhibition on dopaminergic neurons in pathogenic conditions require further elucidation, and may provide a potential efficient target for treating PD by blocking the pathogenic pathway.

## Abbreviations

GSK-3: glycogen synthase kinase-3
PD: Parkinson’s disease
LBs: Lewy bodies
SN: substantia nigra
mPTP: mitochondrial permeability transition pore
MPTP: 1-methy-4-phenyl-1,2,3,6-tetrahydropyridine
MPP^+^: 1-methyl-4-phenylpyridinium
ROS: reactive oxygen species
TNF-α: tumor necrosis factor-α
